# Multi-Omics Analysis of Cancer Cell Lines with High/Low Ferroptosis Scores and Development of a Ferroptosis-Related Model for Multiple Cancer Types

**DOI:** 10.3389/fcell.2021.794475

**Published:** 2021-12-06

**Authors:** Guangyao Shan, Huan Zhang, Guoshu Bi, Yunyi Bian, Jiaqi Liang, Besskaya Valeria, Dejun Zeng, Guangyu Yao, Cheng Zhan, Hong Fan

**Affiliations:** Department of Thoracic Surgery, Zhongshan Hospital, Fudan University, Shanghai, China

**Keywords:** ferroptosis, cell line, drug resisitance, multi-omics, pan-cancers

## Abstract

**Background:** Ferroptosis is a newly identified regulated cell death characterized by iron-dependent lipid peroxidation and subsequent membrane oxidative damage, which has been implicated in multiple types of cancers. The multi-omics differences between cancer cell lines with high/low ferroptosis scores remain to be elucidated.

**Methods and Materials:** We used RNA-seq gene expression, gene mutation, miRNA expression, metabolites, copy number variation, and drug sensitivity data of cancer cell lines from DEPMAP to detect multi-omics differences associated with ferroptosis. Based on the gene expression data of cancer cell lines, we performed LASSO-Logistic regression analysis to build a ferroptosis-related model. Lung adenocarcinoma (LUAD), lung squamous cell carcinoma (LUSC), esophageal cancer (ESCA), bladder cancer (BLCA), cervical cancer (CESC), and head and neck cancer (HNSC) patients from the TCGA database were used as validation cohorts to test the efficacy of this model.

**Results:** After stratifying the cancer cell lines into high score (HS) and low score (LS) groups according to the median of ferroptosis scores generated by gene set variation analysis, we found that IC50 of 66 agents such as oxaliplatin (*p < 0.001*) were significantly different, among which 65 were higher in the HS group. 851 genes such as KEAP1 and NRAS were differentially muted between the two groups. Differentially expressed genes, miRNAs and metabolites were also detected—multiple items such as IL17F (logFC = 6.58, *p < 0.001*) differed between the two groups. Unlike the TCGA data generated by bulk RNA-seq, the gene expression data in DEPMAP are from pure cancer cells, so it could better reflect the traits of tumors in cancer patients. Thus, we built a 15-signature model (AUC = 0.878) based on the gene expression data of cancer cell lines. The validation cohorts demonstrated a higher mutational rate of NFE2L2 and higher expression levels of 12 ferroptosis-related genes in HS groups.

**Conclusion:** This article systemically analyzed multi-omics differences between cancer cell lines with high/low ferroptosis scores and a ferroptosis-related model was developed for multiple cancer types. Our findings could improve our understanding of the role of ferroptosis in cancer and provide new insight into treatment for malignant tumors.

## Introduction

Ferroptosis is a newly identified regulated cell death (RCD) characterized by iron-dependent lipid peroxidation and subsequent membrane oxidative damage (Stockwell et al., 2017). Another type of RCD, apoptosis, has been perceived as the only form of RCD suitable for developing anti-tumor therapies for a long time (Liang et al., 2019). While the effect of agents targeted apoptosis is not as good as expected owing to the multiple mechanisms cancer cells have developed to resist cell death. Distinct from apoptosis, ferroptosis has its unique molecular mechanisms. Ferroptosis can be induced *via* extrinsic or intrinsic pathways. The extrinsic pathway is initiated by the blockage of cell membrane transporters such as the cystine/glutamate transporter (also known as system xc^−^) or by activation of the iron transporters serotransferrin and lactotransferrin. The intrinsic pathway is triggered by inhibiting intracellular antioxidant systems such as glutathione-glutathione peroxidase 4 (GSH-GPX4) (Lu et al., 2017).

Compared with normal cells, the growth of cancer cells (especially cancer stem cells) strongly depends on iron. The pretreatment of erastin, an inducer of ferroptosis, could synergize with cisplatin (a GSH inhibitor and interfering agent of DNA replication) to magnify its anti-tumor effects (Sato et al., 2018). Likewise, multiple ferroptosis inducers, such as Sorafenib and Altretamine, could serve as radiosensitizers *via* inhibiting SLC7A11 (a protein controlling the transport of cystine and glutamate) or GPX4 activities (Lei et al., 2021). It could be concluded that ferroptosis provides new strategies to kill tumor cells alone or in combination with other conventional therapies. Given the promising potential of ferroptosis in cancer treatment, it’s imperative to elucidate the multi-omics differences influenced by this pathway.

In this article, we systemically analyzed the multi-omics differences between the cancer cell lines from the Dependency Map (DEPMAP) with high/low ferroptosis scores generated by gene set variation analysis (GSVA). Unlike the bulk RNA-seq data from the cancer genome atlas (TCGA), the gene expression data in DEPMAP are only from cancer cells, which could better reflect the traits of tumors in cancer patients. Therefore, we built a Least absolute shrinkage and selection operator (LASSO)-Logistic model based on the gene expression data of cancer cell lines to classify the cancer patients into HS and LS groups. Finally, we validated this model in bladder cancer (BLCA), cervical cancer (CESC), esophageal cancer (ESCA), head and neck cancer (HNSC), lung adenocarcinoma (LUAD), and lung squamous cell carcinoma (LUSC) patients from TCGA. We hope our study could improve our understanding of the role of ferroptosis in cancer, thus shedding new light on the development of new regimens.

## Materials and Methods

### Data Collection and Processing

Data of cancer cell lines, including sample information, RNA-seq gene expression, gene mutation, miRNA expression, metabolites, copy number variation (CNV), and drug sensitivity IC50 (Sanger GDSC2), were obtained from DEPMAP ( *https://depmap.org/portal/* ). After matching the gene expression, gene mutation, miRNA expression, and CNV data with sample information, 1376 gene expression, 940 miRNA, 911 metabolites, 1295 gene mutation, 1356 CNV, and 595 drug sensitivity data were included for further analysis.

For the 33 kinds of cancer types in TCGA ( *https://portal.gdc.cancer.gov/* ), ones that complied with the following two standards were used as validation cohorts for the ferroptosis-based classification model: 1) The RNA-seq gene expression data, somatic mutation data, and survival information of the patients were intact. 2) The number of patients in the HS or LS group was greater than 10, respectively. 400 BLCA (23 HS *vs*. 377 LS), 283 CESC (11 HS *vs*. 272 LS), 151 ESCA (40 HS *vs*. 111 LS), 494 HNSC (50 HS *vs*. 444 LS), 497 LUAD (45 HS *vs*. 452 LS), and 489 LUSC (162 HS *vs*. 372 LS) patients were included in this study.

### GSVA and Drug Sensitivity

108 driver genes that promote ferroptosis and 69 suppressor genes preventing ferroptosis were obtained from the FerrDb (Zhou and Bao, 2020) ( *http://www.zhounan.org/ferrdb/* ). After removing the duplicated ones, 173 ferroptosis-related genes were recruited for further analysis. Besides ferroptosis, multiple biological processes (Supplementary file S1) were also detected. By integrating the collective expression of the given gene sets above, the enrichment scores of 76 biological pathways for 1376 cancer cell lines were obtained *via* an unsupervised gene set enrichment method in the R *GSVA* package. Instead of ssGSEA, the GSVA method is preferred in our study because GSVA includes the normalization of gene expression to reduce the noise of the data and has been shown to outperform ssGSEA when measuring the signal-to-noise ratio in differential gene expression and differential pathway activity identification analyses (Bi et al., 2020). According to the median value of the GSVA score of ferroptosis, the cancer cell lines were stratified into the high score group (HS) and low score group (LS). To further explore the relationship between drug sensitivity and ferroptosis, we compared the IC50 of 169 agents between the two groups.

### Differentially Muted Genes

Gene mutation data of cancer cell lines were separated into HS and LS groups. Next, the R package *ComplexHeatmap* was used to visualize the different mutation patterns of the two groups. The two-sided Fisher exact test was used to determine the DMGs between the two groups, and *p* < 0.05 was considered significant. Besides, mutational load and CNV were calculated for every cancer cell line and subsequently compared between the two groups.

### Differentially Expressed Genes, miRNAs, Metabolites, and Enrichment Analysis

Based on the RNA-seq gene expression data of cancer cell lines, DEGs were investigated by the R *edgeR* package. The absolute value of Log_2_FoldChange (|Log FC |) >1 and *p*-value <0.05 were considered as significantly different. Subsequently, Gene Ontology (GO) and Kyoto Encyclopedia of Genes and Genomes (KEGG) enrichment analyses were performed using R *org.Hs.eg.db* and *Clusterprofiler* packages. Similarly, different miRNA and metabolite expression patterns were also explored between the two groups. |Log FC | >0.5 and *p*-value <0.05 were considered as significantly different for miRNAs. The criteria for defining differentially expressed metabolites were set as follows: |Log FC| >1 and *p*-value <0.05.

### Protein-To-Protein Interaction Network

To further investigate the inner correlation of the DEGs, we mapped the top 100 most significantly upregulated and downregulated DEGs in STRING (*version 11.0b,* *https://www.string-db.org/* ) to make a PPI network. Next, the Molecular Complex Detection (MCODE) plugin (Bader and Hogue, 2003) in Cytoscape (*version 3.8.2*) was used to detect modules of the PPI network (degree cutoff = 2, node score cutoff = 0.2, k-score = 2, and max depth = 100).

### Construction of the Ferroptosis-Related Model

Compared to the gene expression data from TCGA generated by bulk RNA-seq, data in DEAPMAP are pure cancer cells, which could better reflect the traits of tumor cells in cancer patients. Therefore, they are more appropriate to build the ferroptosis-related classification model. Based on the gene expression data in DEPMAP, we developed a ferroptosis-related model to stratify the cancer cell lines into HS and LS groups. Firstly, we take the intersection of 173 ferroptosis-related genes and DEGs, and 22 genes were recognized as ferroptosis-related DEGs. Based on the 22 candidate genes, we performed LASSO and binary Logistic regression analysis to construct the model. LASSO is a penalized method to select data with high dimensions and reduce the impact of overfitting (Vasquez et al., 2016). Ten-fold cross-validation was adopted using the R *glmnet* package to determine the optimal parameter λ and corresponding genes. Fifteen genes were enrolled by LASSO regression analysis, and their coefficients were decided by binary logistic regression analysis. Harrell’s concordance index (C-index) was used to evaluate the accuracy of the classification model.

### Validation of the Ferroptosis-Related Model

The BLCA, CESC, ESCA, HNSC, LUAD, and LUSC patients from TCGA were stratified into HS and LS groups according to the cutoff value of the classification model. Ferroptosis-associated DMGs, ferroptosis-related gene expression patterns, and Kaplan–Meier (K-M) survival analysis were investigated between the two groups.

### Statistical Analyses

All statistical analyses were carried out in R software (Version 4.1.1, see in Data Availability Statement). The comparison of the baseline characteristics of cancer cell lines from different groups was conducted, of which categorical variables were compared by Chi-square test or Fisher exact test when appropriate and continuous variables were compared by Student’s t-test. The Student’s t-test was also used to compare continuous variables such as the CNV, mutation load, IC50 of agents. K-M survival curves were visualized by the R *ggplot2* package, and the log-rank test was used to compare the overall survival between the two groups. All the *p* values were two-sided, and the significance threshold for *p*-value in all tests was 0.05.

## Result

### GSVA and Drug Sensitivity

**FIGURE 1:**
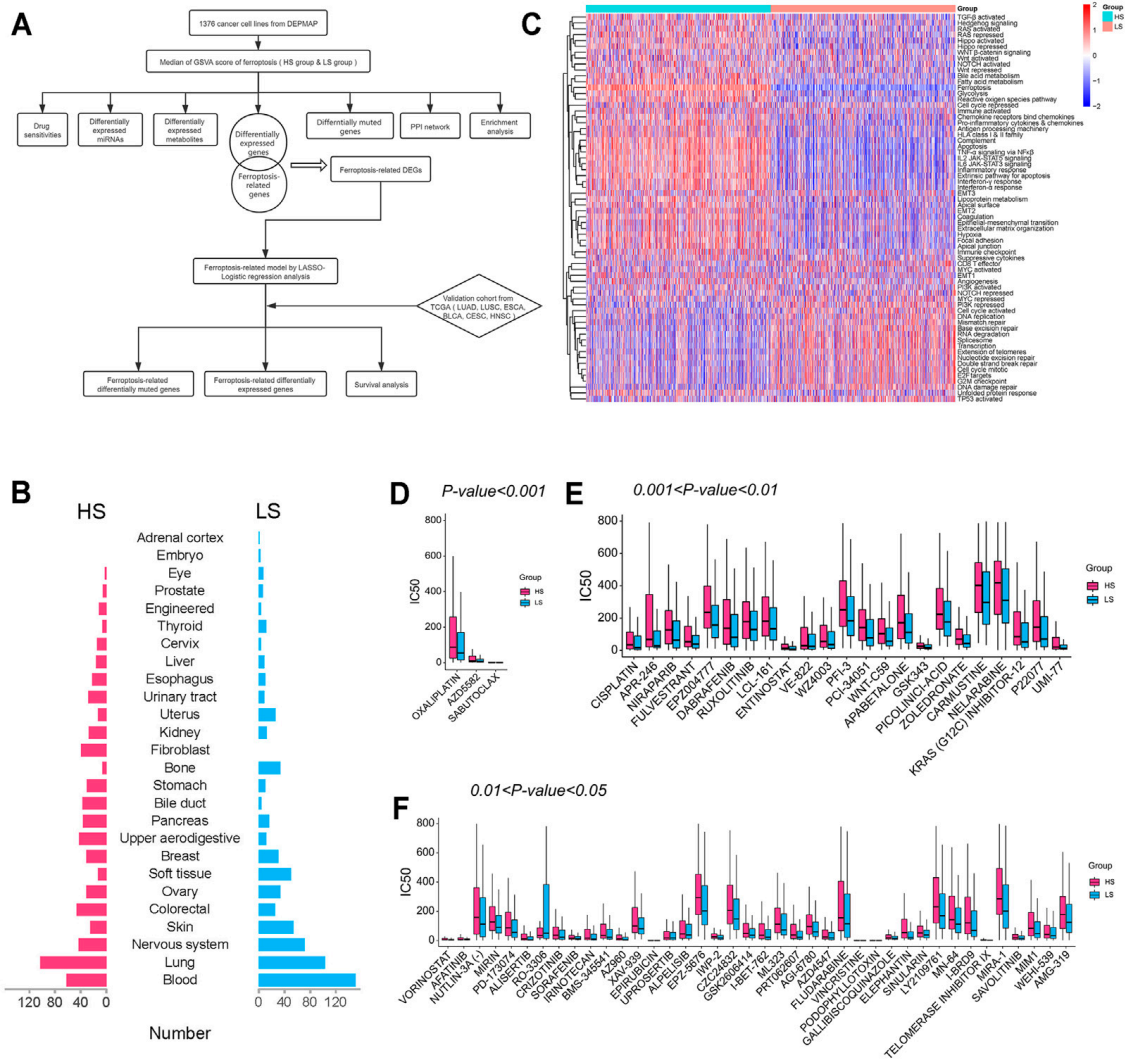
**(A)** Flow diagram of the whole design. **(B)** Tissue source of cancer cell lines included in this study. **(C)** Heatmap of gene set variation analysis (GSVA) of cancer cell lines. **(D–F)** IC50 of agents with different thresholds of significance between the HS and LS groups.

The whole design of this study is shown in Figure 1A. The cancer cell lines (Figure 1B) were divided into the HS (*n* = 688) and LS (*n* = 688) groups according to the median of the ferroptosis score generated by GSVA. Beside ferroptosis, the two groups differed in multiple biological ways, such as epithelial-mesenchymal transition and cell cycle activated (Figure 1C).

After comparing the IC50 of 169 drugs between the two groups, we found 66 of them were significantly different (Figures 1D–F). Among them, oxaliplatin (460.3 HS *vs.* 333.2 LS, μM/L, *p < 0.001*) is the most significantly different drug, and vincristine (21.3 HS *vs.* 2.8 LS, μM/L, *p < 0.05*) is the agent with the greatest fold change. Except for RO-3306 (60.8 HS *vs.* 201.2 LS, μM/L, *p < 0.05*), all agents with a significant difference have a higher IC50 in the HS group, indicating patients with a high ferroptosis score may be less likely to benefit from these chemotherapeutic and targeted drugs. For example, the IC50 of cisplatin (477.0 HS *vs.* 211.4 LS, μM/L, *p < 0.01*), afatinib (35.3 HS *vs.* 15.8 LS, μM/L, *p < 0.01*), and crizotinib (124.8 HS *vs.* 73.1 LS, μM/L, *p < 0.01*) were lower in the LS group; thus lung cancer patients with a low ferroptosis score may be more likely to acquire better clinical outcomes when administrated these agents.

### DMGs

**FIGURE 2:**
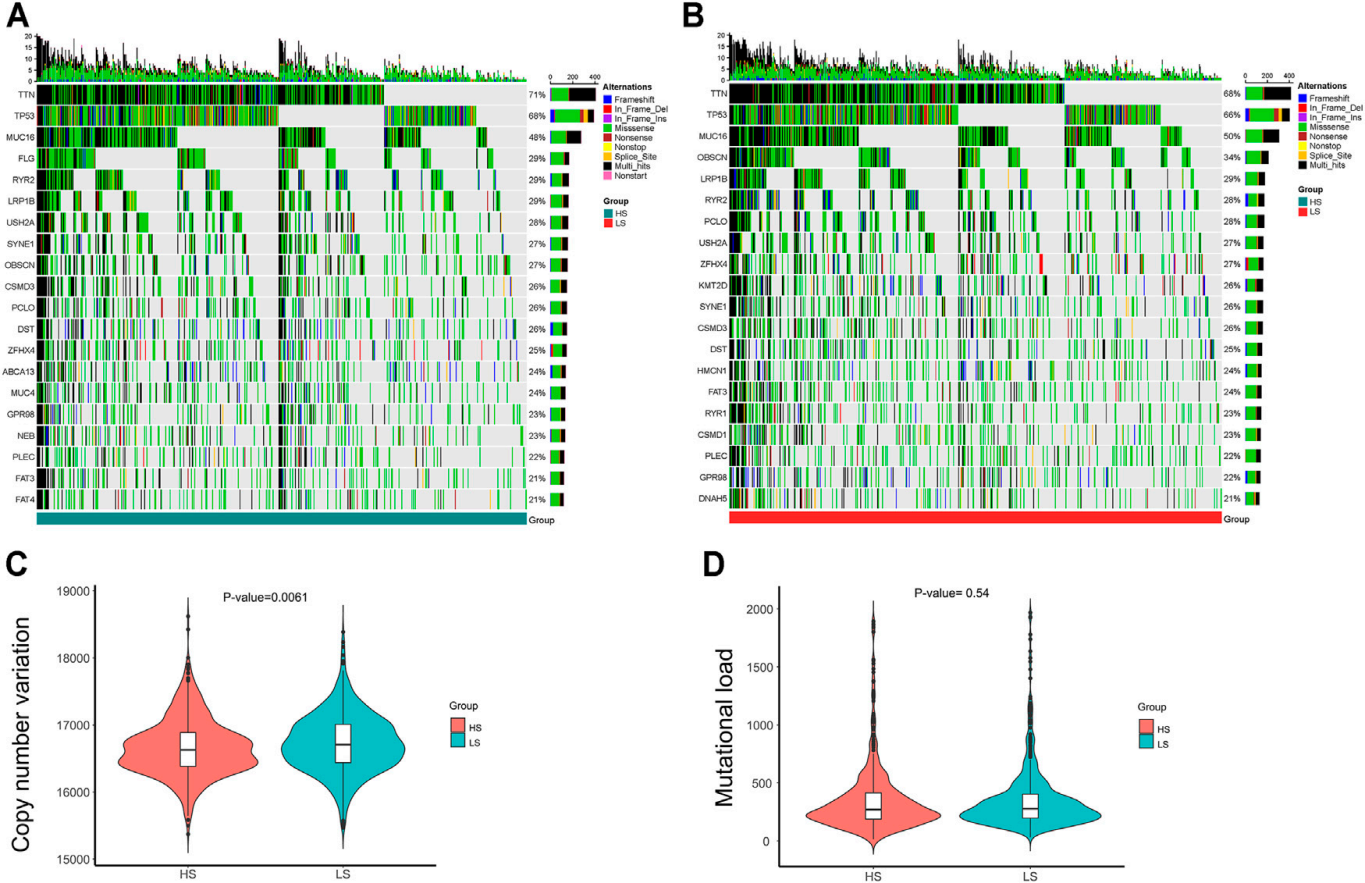
**(A–B)** Top 20 muted genes in the HS and LS groups, separately. **(C–D)** Copy number variation (CNV) and mutational load between the HS and LS groups.

Different mutational patterns of the two groups are shown in Figures 2A,B. Among the 18,725 genes analyzed, 851 were recognized as DMGs, and nine of them such as KEAP1(37% HS *vs.* 17% LS, *p < 0.01*), NRAS (18% HS *vs.* 43% LS, *p < 0.001*), and PROM2 (23% HS *vs.* 14% LS, *p < 0.05*) were related to ferroptosis. Next, we detected the CNV and mutational load (Figures 2C,D) between the two groups. The result suggested the LS group had a higher level of CNV (*p < 0.01*), while the difference of mutational load was not significant.

### DEGs, miRNAs, Metabolites, and Enrichment Analysis

**FIGURE 3:**
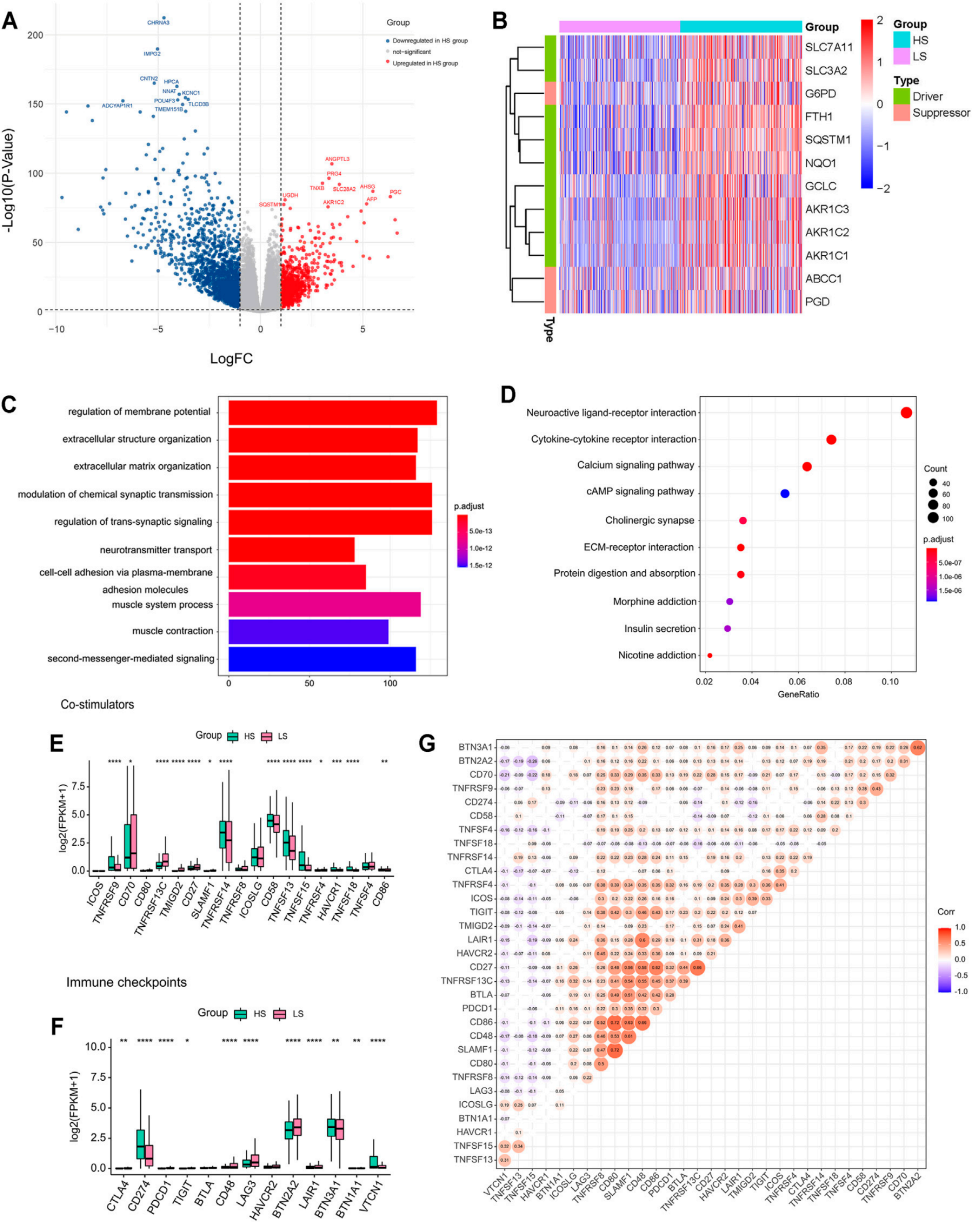
**(A)** Differentially expressed genes (DEGs) between the HS and LS groups. **(B)** Heatmap of 12 ferroptosis-related genes between the HS and LS groups. **(C)** Gene Ontology (GO) enrichment analysis of the biological process of the DEGs. **(D)** Kyoto Encyclopedia of Genes and Genomes (KEGG) enrichment analysis of the DEGs. **(E,F)** Comparison of the expression levels of immune checkpoints and co-stimulators between the HS and LS groups. **(G)** Correlation analysis of the immune checkpoints and co-stimulators. Labels in the circles indicated the correlation coefficients of the corresponding variables. Correlation coefficients without significant levels were hidden. *, p < 0.05; **, p < 0.01; ***, p < 0.001; ****, p < 0.0001.

Among the 16,383 genes analyzed, 868 were upregulated, and 1628 were downregulated in the HS group (Figure 3A). NEUROD6 (logFC = -9.70, *p < 0.001*), PHOX2B (logFC = −9.50, *p < 0.001*), and FSHB (logFC = −8.90, *p < 0.001*) were the most significantly downregulated genes. CRP (logFC = 6.68, *p < 0.001*), IL17F (logFC = 6.58, *p < 0.001*), and PGC (logFC = 6.35, *p < 0.001*) were the most significantly upregulated genes. The expression levels of 12 genes related to ROS detoxification, GSH, and iron metabolism in ferroptosis are shown in Figure 3B; most of them were observed with higher expression levels in the HS group. For miRNAs, 22 were upregulated, and 15 were downregulated among 940 miRNAs (Supplementary Figure S1A). Besides, we also compared metabolites between the two groups, among which 56 were upregulated, and 55 were downregulated in the HS group (Supplementary Figure S1B). Methylnicotinamide (logFC = 19.83, *p < 0.001*) and lactose (logFC = 7.91, *p < 0.001*) were the most significantly upregulated metabolites. Deoxycytidine (logFC = −10.67, *p < 0.001*) and cytidine (logFC = −9.11, *p < 0.001*) were the most significantly downregulated ones.

Next, we used the DEGs to perform GO and KEGG analysis (Figures 3C,D). The GO analysis showed these DEGs were closely related to biological pathways such as regulation of membrane potential and extracellular structure organization. KEGG enrichment analysis indicated items such as neuroactive ligand-receptor interaction, cytokine-cytokine receptor interaction, and calcium signaling pathway differed between the two groups.

Finally, the expression level of the common co-stimulators and immune checkpoint inhibitors (Bian et al., 2021) were compared between the two groups (Figures 3E,F). Most of them were positively correlated (Figure 3G) and differentially expressed. The HS group has a higher expression level of CD274 (*p < 0.0001*), suggesting cancer patients with a high ferroptosis score may be more likely to benefit from PD-L1 inhibitors.

### PPI Network

**FIGURE 4:**
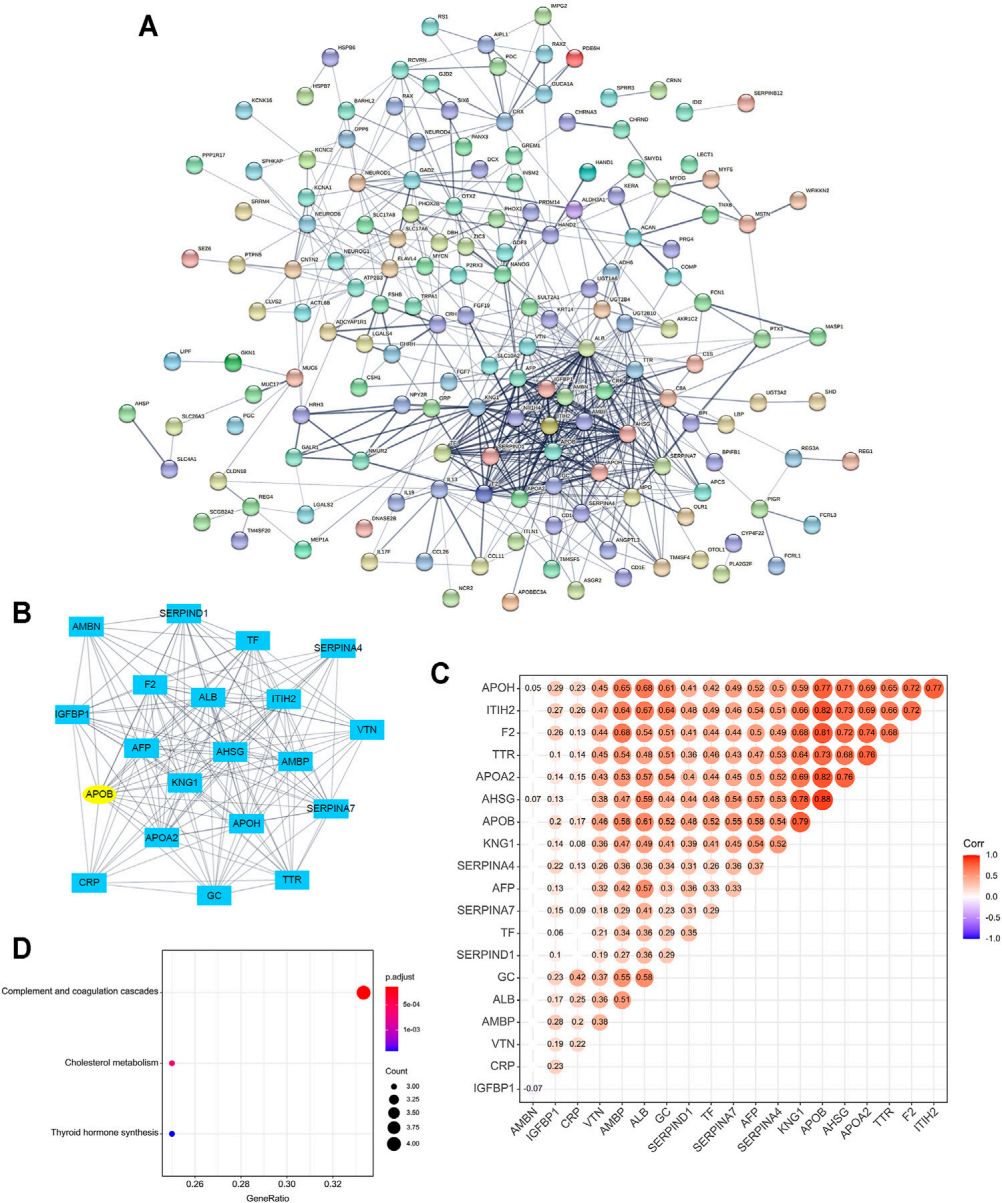
**(A)** Protein-to-protein interaction (PPI) network of the top 200 most significantly upregulated and downregulated genes. The minimum required interaction score is set as 0.400 (medium confidence). Line thickness indicates the strength of data support. **(B)** The cluster of genes with the highest clustering score in the PPI network. **(C,D)** Correlation and KEGG enrichment analysis of the genes with the highest clustering score.

Based on the 200 DEGs, a PPI network was made to elucidate their inner interactions (Figure 4A). The cluster with the highest clustering score (Score = 16.632, Nodes = 20, Edges = 158) demonstrated that 20 genes, including AMBN, SERPIND1, TF, SERPINA4, IGFBP1, F2, ALB, ITIH2, APOB, AFP, AHSG, AMBP, VTN, KNG1, APOA2, APOH, SERPINA7, CRP, GC, and TTR play an important role in the PPI network (Figure 4B). The *Pearson* correlation test showed most of them were positively correlated with significant differences (Figure 4C). KEGG enrichment analysis showed these 20 genes were relevant to complement and coagulation cascades and cholesterol metabolism (Figure 4D).

### Construction of the Ferroptosis-Related Model

In LASSO analysis, the optimal parameter λ was set as lambda.min criteria, which means the minimum binomial deviance (Supplementary Figures S2A,B). Fifteen genes were included in the model, including NOX1, CD44, TP63, NOX3, EPAS1, MYB, CDO1, DUOX2, EGFR, SLC7A11, AKR1C2, PROM2, SQSTM1, CYBB, FTH1 (Supplementary Figure S2C). NOX1, NOX3, EPAS1, MYB, CDO1, DUOX2, EGFR, and CYBB were ferroptosis promoters, and the remaining ones were ferroptosis suppressors. Based on these candidate genes, the model built by binary logistic regression analysis is as follows: score= (0.3025*expression level of NOX1) + (0.1184* expression level of CD44) + (0.0898* expression level of TP63) + (0.2861* expression level of NOX3) + (0.2285* expression level of EPAS1) + (0.2573* expression level of MYB) + (0.1948* expression level of CD O 1) + (0.0747* expression level of DUOX2) + (0.1221* expression level of EGFR) + (0.4443* expression level of SLC7A11) + (0.1399* expression level of AKR1C2) + (0.0520* expression level of PROM2) + (0.4473* expression level of SQSTM1) + (0.1503* expression level of CYBB) + (0.3022* expression level of FTH1). The calibration curve showed the bias-corrected curve and the ideal line almost overlapped, indicating this model has a satisfying classification power (Supplementary Figure S2D). The receiver operating curve (ROC) showed the area under the curve (AUC) was 0.878, and the cutoff value was −0.072 (Supplementary Figure S2E). If the score is greater than this value, it will be stratified as the HS group; otherwise, the LS group.

### Validation of the Ferroptosis-Related Model

**FIGURE 5:**
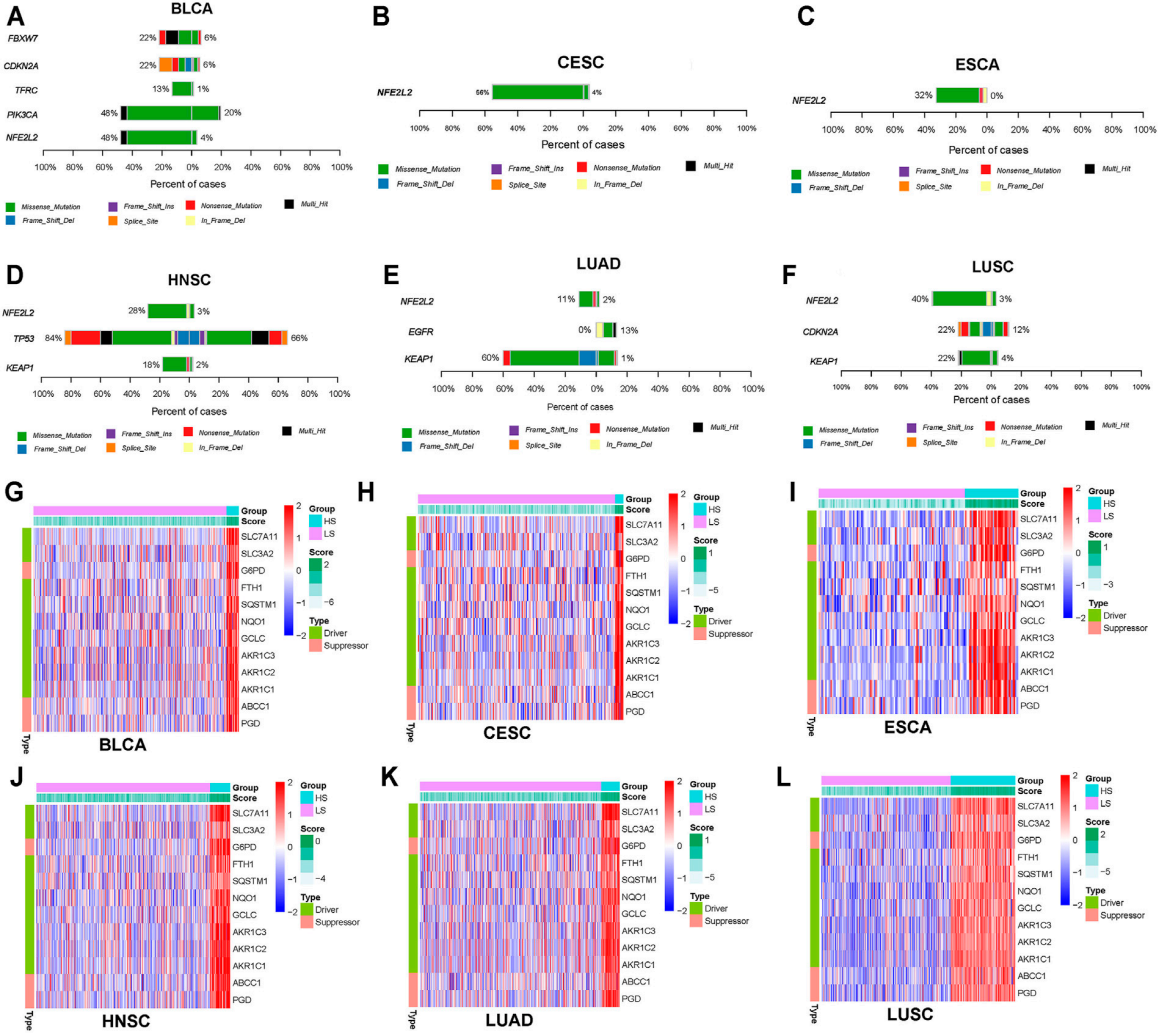
**(A–F)** Ferroptosis-related differentially muted genes between the HS and LS groups in bladder cancer (BLCA), cervical cancer (CESC), esophageal cancer (ESCA), head and neck cancer (HNSC), lung adenocarcinoma (LUAD), and lung squamous cell carcinoma (LUSC) patients. The left side of each figure indicated the HS group; the right side of each figure indicated the LS group. **(G–L)** Heatmap of the fifteen ferroptosis-related genes between the HS and LS groups in BLCA, CESC, ESCA, HNSC, LUAD, and LUSC. Driver indicates gene that promotes ferroptosis; suppressor indicates gene that inhibits ferroptosis.

After dividing the LUAD, LUSC, ESCA, BLCA, CESC, and HNSC patients into HS and LS groups according to the classification model, we explored the ferroptosis-related DMGs between the two groups (Figures 5A–F). NFE2L2, which is associated with response to oxidative stress, was observed with a higher mutational rate in HS groups of all validation cohorts (BLCA, 48% HS *vs* 4% LS, *p < 0.0001*; CESC, 56% HS *vs* 4% LS, *p < 0.0001*; ESCA, 32% HS *vs* 4% LS, *p < 0.0001*; HNSC, 28% HS *vs* 3% LS, *p < 0.0001*; LUAD, 11% HS *vs* 2% LS, *p < 0.01*; LUSC, 40% HS *vs* 3% LS, *p < 0.0001*).

Next, the expression levels of 12 ferroptosis-related genes (GCLC, AKR1C3, AKR1C2, AKR1C1, SLC7A11, SLC3A2, G6PD, FTH1, SQSTM1, NQO1, ABCC1, and PGD) were detected between the two groups. Roughly in accordance with the distribution in cancer cell lines, the HS groups in all validation cohorts had a higher expression level of these ferroptosis-related genes than the LS groups (Figures 5G–L).

**FIGURE 6:**
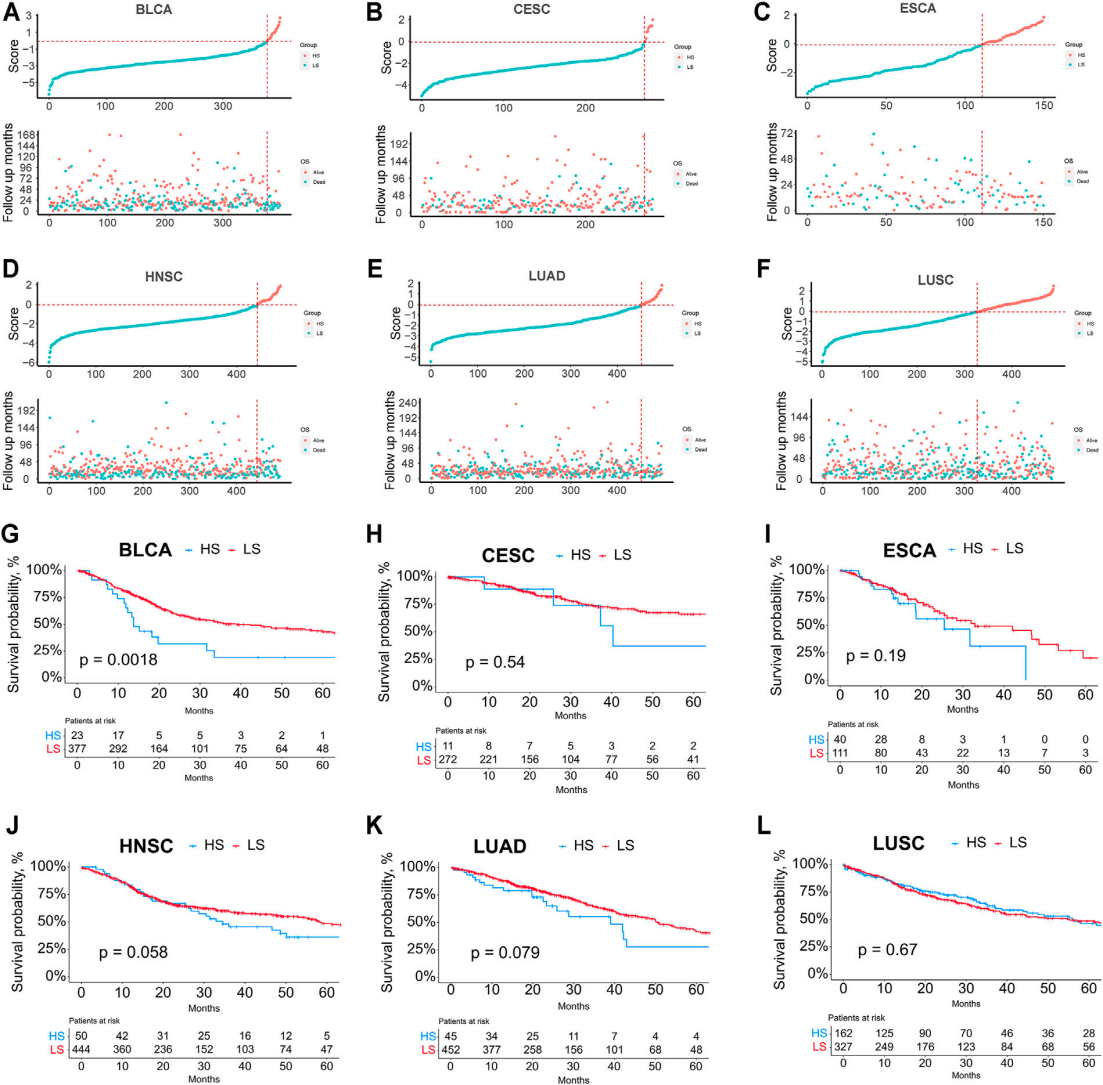
**(A–F)** Relationship between the risk score rank and survival time/survival status in BLCA, CESC, ESCA, HNSC, LUAD, and LUSC. **(G–L)** Kaplan-Meier survival analysis between the HS and LS groups in BLCA, CESC, ESCA, HNSC, LUAD, and LUSC.

Finally, we explored the relationship between the ferroptosis score and survival (Figure 6). K-M survival analysis showed that the HS groups in BLCA, CESC, ESCA, HNSC, and LUAD had poorer survival than the LS groups; however, the difference was not significant except for BLCA.

## Discussion

This study systematically analyzed the multi-omics differences between the cancer cell lines with high/low ferroptosis scores. Multiple biological pathways such as epithelial-mesenchymal transition and drug sensitivities were significantly different between the HS and LS groups. Among 18725 genes analyzed, 851 were defined as DMGs, indicating they may play an important role in the ferroptosis pathway. Compared to the LS group, 868 protein-coding genes such as CRP and IL17F were upregulated in the HS group. In the PPI network, 20 genes, including APOB were recognized as hub genes. Based on the gene expression data of the cancer cell lines, we subsequently built a ferroptosis-related model which could predict the ferroptosis-related gene mutation and expression levels of specific ferroptosis-related genes.

As is shown in Figure 1B, multiple pathways such as epithelial-mesenchymal transition and cell cycle activated differed between the HS and LS groups, suggesting the ferroptosis pathway may have a significant influence on quite a few biological processes. Subsequently, the IC50 of 169 agents was investigated, 66 of which were found significantly different between the two groups. Except for RO-3306 (60.8 HS *vs.* 201.2 LS, μM/L, *p < 0.05*), the IC50 of the remaining 65 agents was higher in the HS group. Oxaliplatin (460.3 HS *vs.* 333.2 LS, μM/L, *p < 0.001*), the most significantly different agent, is a third-generation platinum drug widely used in the treatment of colorectal cancer (CRC). However, less than 40% patients with advanced CRC could benefit from oxaliplatin due to the development of drug resistance (Hsu et al., 2018). A recent study has shown that congenital or acquired oxaliplatin resistance in CRC cell lines could be reversed by the treatment of RSL3 (a ferroptosis inducer) (Yang et al., 2021). Another broad-spectrum anti-cancer drug, vincristine (21.3 HS *vs.* 2.8 LS, μM/L, *p < 0.05*), also inevitably falls into the dilemma of drug resistance after a period of usage (Xia et al., 2018). It has been elucidated that vincristine could significantly increase the expression of LINC00618, which could promote ferroptosis *via* inhibiting SLC7A11 (Wang et al., 2021). Besides, it’s notable that the HS group has a higher expression level of CD274 (*p < 0.0001*), indicating cancer patients with a high ferroptosis score may be more likely to benefit from PD-L1 inhibitors. In general, ferroptosis plays a critical part in the effect and resistance acquisition of multiple anti-cancer drugs. The combination use with ferroptosis inducers may improve the clinical benefit of chemotherapeutic and targeted agents for cancer patients. The role of ferroptosis in drug resistance sheds new light on the development of new regimens, but it still needs further investigation in experiment and clinical trials.

C-reactive protein, which is encoded by the CRP gene, is well-known in the acute-phase inflammatory response. Identified as a hallmark of cancer, tumor-promoting inflammation is proven to lead to multiple hallmark capabilities acquisition, such as sustaining proliferative signals by providing some bioactive substance into the tumor microenvironment (Hanahan and Weinberg, 2011). As an indicator of inflammatory levels, patients who harbor a high level of CRP postoperatively are more likely to suffer from more complications and increased mortality (Jones et al., 2006; Lopez-Pastorini et al., 2017). The DEG analysis has shown the CRP is upregulated (logFC = 6.68, *p < 0.001*) in the HS group, suggesting patients with a high ferroptosis score may have a higher level of inflammation and poorer survival.

In the PPI network, 20 genes were recognized as hub genes, which were closely related to the cholesterol metabolism by KEGG analysis. Studies in both cell and animal levels revealed that cholesterol synthesis induced by the AKT/mTORC1/SREBP pathway could lead to cell growth and promoted cancer aggressiveness and bone metastases. Besides, numerous cholesterol metabolites such as steroids were found to favor tumor growth and metastasis (Deng et al., 2020). APOB (logFC = 3.41, *p < 0.001*) functions as the seed gene in the hub cluster of genes. Apolipoprotein B (ApoB) is an amphipathic glycoprotein that plays a central role in human lipoprotein metabolism, the mutation of which could either cause hypercholesterolemia or hypobetalipoproteinemia (Whitfield et al., 2004). Since ferroptosis is characterized by excessive lipid peroxidation, this process may be indirectly influenced by ApoB. Few pieces of research focused on the effect of APOB in ferroptosis; thus, its role in ferroptosis still needs further investigation.

Typically, GSVA is more suitable for a rather large group of subjects to classify them into different groups. For a single patient or a small group of patients, GSVA may not be appropriate. Therefore, we developed a LASSO-Logistic model to identify the ferroptosis status of a single patient or a small group of patients by employing just 15 ferroptosis-related genes. Unlike the TCGA data generated by bulk RNA-seq, the gene expression data in DEPMAP are only from cancer cells, so this model could better reflect the traits of tumors in cancer patients. Since the model was generated from pure cancer cells, it may be more suitable for the analysis of cancer cells. However, it is not easy to get pure cancer cells in clinical settings. Through analysis, we found this model could also be used in the bulk RNA-seq data of entire tumor samples like TCGA.

Compared to the LS groups, the HS groups of all validation cohorts harbored a significantly higher mutational rate of NFE2L2, which is a ferroptosis suppressor that can protect cells from oxidative damage (Kuang et al., 2020). As a critical transcription factor in ferroptosis, multiple genes were regulated by NFE2L2. GSS, GCLC SLC7A11, and GPX4, which are NFE2L2-dependent genes, play an important role in the synthesis and function of GSH-GPX4 antioxidation system (Dai et al., 2020; Tang et al., 2021). What’s more, iron metabolism-associated genes such as FTH1, FTL, and SLC40A1 also have a close relationship with NFE2L2. Among them, FTH1-FTL complex is responsible for iron storage and could prevent Fe^2+^ from being oxidated; SLC40A1 is in charge of iron export from cells (Chen et al., 2021). Recent studies suggested that aldo-keto reductases (AKRs), a superfamily of NADPH-linked oxidoreductases, including AKR1C1, AKR1C2, and AKR1C3, are potential NFE2L2 target genes responsible for ferroptosis resistance via inhibiting lipid peroxidation in melanoma cells (Dai et al., 2020). Given the key role of NFE2L2 in ferroptosis, it may be a promising target for drug development for treating malignant tumors.

The HS groups of all the five cancer types tested had a higher expression level of the 12 ferroptosis-related genes (GCLC, AKR1C3, AKR1C2, AKR1C1, SLC7A11, SLC3A2, G6PD, FTH1, SQSTM1, NQO1, ABCC1, and PGD). Notably, about half of them could be regulated by NFE2L2. GO enrichment analysis suggested these genes were related to response to oxidative stress *(adjusted p-value < 0.001)* and glutathione metabolism (*adjusted p-value < 0.001*)*,* both of which are critical biological processes in ferroptosis. It could be concluded from the distribution of these ferroptosis-associated genes that the model had a superior discrimination power.

The relationship between the groups stratified by the model and prognosis was vague, partially attributed to the insufficient number of patients in the HS group. Although the significant difference in survival analysis could only be seen in BLCA, the HNSC and LUAD cohorts also demonstrated a better prognosis in the LS groups with *p*-values slightly greater than 0.05. The correlation between the classification model and prognosis may be further clarified in a larger number of cohorts. It’s likely that a panel consisting of the 15 ferroptosis-related genes may be developed for clinical use to test the ferroptosis status of cancer patients in the future. And we hope the efficacy of this model and the relationship with radiotherapy, chemotherapy, and immunotherapy can be further investigated at that moment.

There are also some limitations of our study. The ferroptosis-related geneset provided by the FerrDb website may not be accurate enough due to insufficient studies about the role of ferroptosis in tumors up to now. Therefore, some important ferroptosis-related genes may be neglected. Besides, the reaction and mechanisms of ferroptosis vary with cancer types. Although we used 1376 cell lines from more than 20 cancer types to build this model and tried to validate it in 33 kinds of cancer types to reach a general conclusion, the efficacy and universality of this ferroptosis-related classification model still needs to be further evaluated a larger outer data and prospective studies. What’s more, as single-cell sequencing may be more widely used in the future, it is promising that obtaining the RNA-seq data of pure tumor cells from a tumor tissue will be easier. We hope this model can be further investigated at single-cell level at that time.

## Conclusion

In a word, we systemically analyzed the multi-omics differences between the cancer cell lines with high or low ferroptosis scores. Based on the gene expression data of multiple cancer cell lines, the LASSO-binary Logistic regression analysis was performed to build a 15-signature model which could predict the expression levels of specific ferroptosis-related genes and ferroptosis-related gene mutation. We hope our research could improve our understanding of the ferroptosis pathway in cancer and provide new insight into treating patients with malignant tumors.

## Data Availability

The original contributions presented in the study are included in the article/Supplementary Material. The working sheets and code used to analyze the data in our study could be obtained by clicking the link (https://www.jianguoyun.com/p/DUk4IxEQituHChjT2p0E), further inquiries can be directed to the corresponding authors.
